# *Staphylococcus aureus* Entrance into the Dairy Chain: Tracking *S. aureus* from Dairy Cow to Cheese

**DOI:** 10.3389/fmicb.2016.01603

**Published:** 2016-10-13

**Authors:** Judith Kümmel, Beatrix Stessl, Monika Gonano, Georg Walcher, Othmar Bereuter, Martina Fricker, Tom Grunert, Martin Wagner, Monika Ehling-Schulz

**Affiliations:** ^1^Department of Pathobiology, Functional Microbiology, Institute of Microbiology, University of Veterinary MedicineVienna, Austria; ^2^Clinic for Ruminants, Department for Farm Animals and Herd Management, University of Veterinary MedicineVienna, Austria; ^3^Department for Farm Animals and Herd Management, Institute of Milk Hygiene, Milk Technology and Food Science, University of Veterinary MedicineVienna, Austria; ^4^Chamber for Agriculture VorarlbergBregenz, Austria

**Keywords:** *Staphylococcus aureus*, dairy chain, mastitis, FTIR spectroscopy, food safety, subtyping, metabolic fingerpriting

## Abstract

*Staphylococcus aureus* is one of the most important contagious mastitis pathogens in dairy cattle. Due to its zoonotic potential, control of *S. aureus* is not only of great economic importance in the dairy industry but also a significant public health concern. The aim of this study was to decipher the potential of bovine udder associated *S. aureus* as reservoir for *S. aureus* contamination in dairy production and processing. From 18 farms, delivering their milk to an alpine dairy plant for the production of smeared semi-hard and hard cheese. one thousand hundred seventy six one thousand hundred seventy six quarter milk (QM) samples of all cows in lactation (*n* = 294) and representative samples form bulk tank milk (BTM) of all farms were surveyed for coagulase positive (CPS) and coagulase negative Staphylococci (CNS). Furthermore, samples from different steps of the cheese manufacturing process were tested for CPS and CNS. As revealed by chemometric-assisted FTIR spectroscopy and molecular subtyping (*spa* typing and multi locus sequence typing), dairy cattle represent indeed an important, yet underreported, entrance point of *S. aureus* into the dairy chain. Our data clearly show that certain *S. aureus* subtypes are present in primary production as well as in the cheese processing at the dairy plant. However, although a considerable diversity of *S. aureus* subtypes was observed in QM and BTM at the farms, only certain *S. aureus* subtypes were able to enter and persist in the cheese manufacturing at the dairy plant and could be isolated from cheese until day 14 of ripening. Farm strains belonging to the FTIR cluster B1 and B3, which show genetic characteristics (t2953, ST8, enterotoxin profile: *sea*/*sed*/*sej*) of the recently described *S. aureus* genotype B, most successfully contaminated the cheese production at the dairy plant. Thus, our study fosters the hypothesis that genotype B *S. aureus* represent a specific challenge in control of *S. aureus* in the dairy chain that requires effective clearance strategies and hygienic measures already in primary production to avoid a potential transfer of enterotoxic strains or enterotoxins into the dairy processing and the final retail product.

## Introduction

*Staphylococcus aureus*, a facultative anaerobic Gram-positive coccus, is an important cause of bovine mastitis and one of the most cost-intensive diseases in the dairy industry (Dufour et al., 2012). Furthermore, enterotoxigenic *S. aureus* strains have the potential to induce food borne intoxications in humans transmitted by dairy products (Le Loir et al., 2003; Jørgensen et al., 2005; Schmid et al., 2009; Ostyn et al., 2010). Staphylococcal enterotoxins (SEs), which cause abdominal cramps, nausea, emesis, and eventually diarrhea, withstand pasteurization as well as thermal processes, and are resistant against human gastrointestinal proteases (Balaban and Rasooly, 2000). Thus, once SEs are formed in food production and processing, these highly stable toxins will not be destroyed or inactivated by common hygienic measures and pose a health risk for the consumers. Most staphylococcal food borne intoxications are caused by food-handler to food contamination during food processing (Asao et al., 2003). Several case studies of SE related food borne outbreak revealed food-handler as the most likely contamination source since the same *S. aureus* strains were isolated from food-handlers, foods, and/or patient specimens (Wei and Chiou, 2002; Gallina et al., 2013; Johler et al., 2013). Beside this classical “human to food contamination route,” several other entrance points of *S. aureus* into the dairy chain have been described (for overview see Stessl et al., 2011). Biofilms formation in dairy equipment as well as insufficient acidification during cheese manufacturing opens *S. aureus* niches for multiplication and efficient contamination of the dairy processing lines (Sharma and Anand, 2002). Especially the first hours in cheese processing are important for control of *S. aureus*. Using three model cheeses, produced from cows' raw milk, maximal levels of *S. aureus* were found after 1 day of ripening and SE production was tightly linked to pH (Delbes et al., 2006).

Furthermore, cows suffering from subclinical mastitis are increasingly discussed as alternative reservoirs of SE producing *S. aureus* contaminating dairy production and processing chains. For instance, a recent food borne outbreak in Austria caused by pasteurized milk products contaminated with SEs could be linked to cows suffering from *S. aureus* mastitis. The cows and not the dairy owner were identified as the most likely reservoir of the enterotoxin producing *S. aureus* (Schmid et al., 2009). Genotype B *S. aureus*, which is usually associated with bovine intermammary infections (IMIs) and known for its high within-herd prevalence of *S. aureus* mastitis (Fournier et al., 2008; Cremonesi et al., 2015), was also reported to be an important source of *S. aureus* contamination of Swiss raw milk cheeses (Hummerjohann et al., 2014). The hypothesis that cows with *S. aureus* IMI infections may indeed represent reservoirs for dairy production chain contamination is further fostered by a recent report from an outbreak at a Swiss boarding school linked to a soft cheese contaminated with *S. aureus*, showing the genetic characteristics of the bovine associated genotype B (Johler et al., 2015). Although the aforementioned reports are pointing toward dairy animals as—so far underestimated—reservoirs for *S. aureus* contaminations of dairy products, a direct proof for the transmission from cow to dairy plants and products is still lacking.

The aim of this study was therefore to decipher the potential of bovine udder associated *S. aureus* as reservoir for *S. aureus* contamination in dairy production and processing. The whole dairy chain from quarter milk samples of individual cows to the retail dairy products was surveyed. Fourier Transform Infrared (FTIR) Spectroscopy was employed as high throughput method for identification and typing of *S. aureus* and related organisms. FTIR spectroscopy is a vibrational spectroscopic technique that allows the identification and discrimination of microorganisms at different taxonomic levels (Naumann et al., 1991; Ehling-Schulz et al., 2005; Wenning and Scherer, 2013). Very recently, a comparative study, including isolates from animal, human, and food sources, revealed a similar discriminatory power of FTIR spectroscopy and molecular fingerprinting methods for typing of *S. aureus* (Johler et al., 2016a), highlighting the potential of FTIR for tracing and tracking *S. aureus* along the dairy production and processing chain from farm to table.

## Materials and methods

### Dairy chain sampling

To gain a better understanding of the entrance points and contamination routes of *S. aureus* in the dairy production chain, a semi-hard cheese (so called “mountain cheese”) production in west of Austria (district Vorarlberg) was sampled from cow to product. Quarter milk (QM) samples, composite milk samples, and bulk tank milk (BTM) were collected during 2 days (morning and evening milk) from 18 dairy farms delivering their milk to one alpine dairy. After storage of the BTM at the dairy for 18 h at 8°C, the semi-hard cheese made from raw milk was processed within 1 day, soaked for up to 2 days in brine and ripened in the ripening cellar for 4 months. The cheese manufacturing was surveyed by taking samples at several cheese processing stages as outlined below.

#### Sampling at the farm level

At the surveyed farms a total of 1176 quarter milk samples from all cows in lactation were collected in addition to the routinely taken composite milk samples from each cow (*n* = 294). The herd size varied from 3 to 43 lactating animals predominately Brown Swiss breed (see Table 1). The average milk yield per cow ranged from 2.91 to 58.28 liters. The mean amount of milk per herd produced in two milking times ranged from 41.70 to 743.10 liters (median: 249.55 liters). QM samples were collected aseptically from foremilk following the guidelines of the National Mastitis Councils Laboratory Handbook on Bovine Mastitis (Hogan et al., 1999). Each quarter of each cow was screened for cell count abnormalities prior to sample collection by using the California Mastitis Test (CMT). Somatic cell counts (SCC) were determined by a Fossomatic milk cell counter (Foss, Hillerød, Denmark) from composite milk samples and BTM samples. Additionally, QM samples were taken aseptically for microbiological examination. The average sample volume per quarter was 5 ml collected in sterile plastic tubes (Sterilin Limited, Cambridge, UK). Collected samples were stored in cool boxes and transported immediately to the laboratory for further investigation. The sterile QM samples were centrifuged and a loop of sediment (10 μl) was streaked onto on Columbia agar supplemented with 5% sheep blood (Thermofisher Scientific Inc., Oxoid Ltd., Hampshire, UK), and on Baird-Parker agar (Oxoid Ltd.). Plates were incubated at 37°C and examined after 24–48 h for bacterial growth. Furthermore, BTM samples were collected at each farm (*n* = 18) aseptically.

**Table 1:** **Farm data comprising Somatic cell counts (SCC), *Staphylococcus* spp. positive cows and bulk tank milk samples**.

**Farm**	**Lactating cows (n)**	**Milk amount (l)^a^**	**Milk amount/cow (l)**	**Ø Herd SCC^b^**	**Ø SCC^c^ of SA pos. cows**	**Individual cow^d^**		**BTM^e^**	
						***S. aureus***	**CNS**	***S. aureus***	**CNS**
1	43	743,1	17,28	228.976	55.000	1	11	−	+
2	32	93,1	2,91	162.531		0	8	−	+
3	17	83	4,88	134.176	181.909	10	1	+	+
4	10	137,7	13,77	141.700		0	0	+	+
5	21	237,9	11,33	222.857		0	2	−	+
6	16	631,6	39,48	233.187	859.500	2	1	+	−
7	25	525,7	21,03	246.840	381.000	1	0	−	+
8	18	127	7,06	169.000	74.000	1	3	+	+
9	6	41,7	6,95	98.166		0	2	−	+
10	6	44	7,33	236.666		0	1	−	+
11	9	436,4	48,49	747.625		0	1	−	+
12	11	261,2	23,75	301.909		0	0	+	+
13	13	104,8	8,06	125.615	110.300	6	0	+	+
14	29	482,2	16,63	168.642	349.500	2	2	+	+
15	22	292,4	13,29	412.363	132.500	2	3	+	+
16	10	518,7	51,87	173.111		0	0	+	+
17	5	291,4	58,28	77.000	23.000	1	0	−	+
18	3	56,8	18,93	69.333		0	0	+	+

a *Average milk amount per milking*.

b *SCC: Average somatic cell count = cells/ml determined on herd level including SCC from all cows*.

c *SCC: Average somatic cell count = cells/ml determined on herd level including SCC from cows tested positive for S. aureus*.

d *Quarter milk samples positive for S. aureus and Coagulase Negative Staphylococci (CNS) per individual cow and farm*.

e *BTM: bulk tank milk samples positive for S. aureus*.

#### Sampling at dairy level

In addition, samples, which are subsumed as “dairy,” were taken during cheese manufacturing at the dairy processing the milk of the aforementioned farms. The sampling of the production of a semi-hard cheese, made from raw milk, included: stored milk in the vat tank, milk before processing, curd, whey, cheese after pressing, brine, cheese during brining, cheese after day 1, 7, 14, 28, 58 of ripening and at retail level. During cheese processing the free water activity dropped from 0.99 (in curd) to 0.92 (end of ripening) while the pH increased from pH 5.1 (first day of ripening) to pH 6.3 (at retail level) (for further details on the cheese manufacturing see Walcher et al., 2014). All samples were transported in cool boxes immediately to the laboratory for further investigation. *Staphylococcus* spp. counts were determined according to ISO 6888-1 (1999). In brief, 25 grams of three subsamples (A, B, C) of each solid sample e.g., curd or cheese at different stages of ripening were diluted 1:10 in 225 ml in sterile Ringer's solution (Oxoid Ltd.), homogenized in a laboratory blender (Stomacher 400, Seward, Worthing, UK) and 100 μl of each samples were surface plated on Baird Parker agar. Liquid samples, such as milk, brine, and whey, were examined directly and in serial dilutions on Baird Parker agar by spatula method. Thirty seven degree for 24–48 h and analyzed after 48 h. Somatic cell counts (SCC) of BTM were determined by using a Fossomatic milk cell counter (Foss, Hillerød, Denmark).

### Bacterial isolation and identification

Up to five presumptive coagulase positive Staphylococci (CPS) and presumptive coagulase negative Staphylococci (CNS) colonies were subcultivated on Tryptone Soya Agar supplemented with 6% yeast (Oxoid Ltd.) for 24–48 h at 37°C. CPS colonies were tested for bound coagulase (clumping factor) by slide coagulase test on sterile microscope slides, and free coagulase by tube coagulase test following ISO 6888-1 (1999) with rabbit plasma (Oxoid, Basingstoke, UK).

The type of hemolysis was recorded on Columbia Sheep Blood agar. Furthermore, the growth potential on BBL CHROMagar MRSA (Becton, Dickinson, and Company, Franklin Lakes, USA) was surveyed. Presumptive *Staphylococcus aureus* colonies were confirmed by *nuc* PCR (Brakstad et al., 1992) and FTIR spectroscopy as outlined below.

### Identification, subtyping and capsule polysaccharide (CP) serotyping by FTIR spectroscopy

CPS and CNS isolates were further investigated by chemometric-assisted FTIR spectroscopy. Its high discriminatory power, low costs and high throughput capacity, make FTIR spectroscopy not only to a valuable method for bacterial identification but also an interesting tool for population studies and epidemiological investigations. It has been shown to be a suitable tool for rapid differentiation of *S. aureus* and Coagulase-Negative Staphylococci (CNS) as well as for the identification and discrimination of bovine mastitis associated gram-positive, catalase-negative cocci and for investigation of the population structure of *Bacillus cereus* (Ehling-Schulz et al., 2005; Lamprell et al., 2006; Schabauer et al., 2014). Sample preparation, FTIR spectroscopic measurements and spectral processing was performed as described previously (Fricker et al., 2010; Grunert et al., 2013). In brief, pure cultured strains were spread by a spatula on tryptone soy agar (Oxoid Ltd.) and incubated at 30°C for 24 h. One loopful of the grown confluent lawn of every strain was diluted in 100 μl distilled water and 30 μl bacteria solution was spotted on a ZnSe sample holder and dried at 40°C for 40 min. Infrared spectra were recorded using a HTS-XT microplate adapter coupled to a Tensor 27 spectrometer (Bruker Optics, Ettlingen, Germany). The OPUS software (version 6.5, Bruker Optics) was used for spectral preprocessing and spectral analysis.

For identification of *S. aureus* spectra obtained from presumptive *S. aureus* colonies were compared to existing FTIR reference spectral libraries, which contain over 7000 strains representing more than 800 species (Wenning et al., 2008). For every identified IR spectra, a hit list containing 10 results was shown arranged in descending order of their *d*-values. *D*-values had to be below 1.5 to be selected for positive identification. The identification result with the lowest *d*-value was selected for positive identification (for details see: Kümmerle et al., 1998; Fricker et al., 2010).

For *S. aureus* subtyping, the highly discriminatory spectral region between 1200 and 800 cm^−1^ dominated by vibrations of various oligo- and polysaccharides was used for hierarchical cluster analysis (HCA) (Johler et al., 2016a). Based on normalized 2nd-derivative spectral data, dendrograms were generated by using the average linkage algorithm with normalization to repro-level 30. Strains were measured once and considered as distinguishable at a spectral distance value > 0.50.

The expression of capsular polysaccharides (CP serotyping) was determined using the previously established artificial neuronal network (ANN) for differential analysis of CP serotype 5 (CP5), CP serotype 8 (CP8), and the CP non-expressing strains (NT) according to Grunert et al. (2013).

### Molecular subtyping, enterotoxin profiling and *mecA* PCR

The *S. aureus* spa typing using the sequence of a polymorphic VNTR in the 3′ coding region of the *S. aureus*–specific staphylococcal protein A (*spa*) was determined as described by Harmsen et al. (2003) (www.spaserver.ridom.de). Spa types were assigned according to the repeat succession with Ridom StaphType TM software (Ridom GmbH, Würzburg, Germany).

Multi-locus sequence typing (MLST) was performed following the protocols of Enright et al. (2000) available on: http://saureus.mlst.net/misc/info.asp. Sequence types (STs) and corresponding clonal complex (CC) were assigned through the MLST database (http://www.mlst.net). *S. aureus* enterotoxin (SE) profiles were determined by employing a multiplex PCR system that targets *sea, seb, sec, sed, seg, seh, sei*, and *sej* (Gonano et al., 2009). PCR based detection of the *mecA* gene was conducted according to Oliveira and de Lencastre (2002).

## Results

### Sampling at farm and dairy level

In frame of this study a dairy production chain was sampled from the cow to the retail product (ripened semi-hard cheese made from raw milk).

At 18 dairy farms that are delivering their milk to an alpine dairy plant for the production of smeared semi-hard and hard cheese, quarter milk (QM) samples (*n* = 1176) from all cows in lactation and representative BTM samples were taken and analyzed for the presence of staphylococci. In addition, composite milk samples from each cow (*n* = 294) and bulk tank (*n* = 18) were taken and the SCC, as an indicator for milk quality and potential udder infection, were determined.

The distribution of *S. aureus* and CNS within the tested farms is depicted in Table 1. In total, 9% of the animals were positive for *S. aureus* and 12% for CNS. *S. aureus* was isolated from 4% (49/1176) of the QM samples and CNS were isolated from 3% (41/1176) of the QM samples. *S. aureus* was found in QM samples from 50% (9/18) of the farms. The number of affected cows on these farms ranged from 1 to 10 *S. aureus* positive animals per herd (6–9%). CNS positive animals were found in 61% (11/18) of the herds with a range of 1–11 animals (0.3–3.7%). *S. aureus* was isolated from 56% (10/18) and CNS from 94% (17/18) of the BTM samples, respectively. Only 33% of the farms with *S. aureus*-positive BTM had also *S. aureus*-positive QM samples (farms 3, 6, 7, 8, 15, 17).

Milk samples from 16 out of the 26 cows tested positive for *S. aureus* showed elevated SCC (≥100,000 cells/ml) but none of these animals showed symptoms of clinical mastitis at the time of sampling. Thus, according to the “*NMC Guidelines on Normal and Abnormal Raw Milk Based on SCC and Signs of Clinical mastitis*” (Smith et al., 2001), *S. aureus* infected cows were classified as subclinical or latent mastitis cases. No significant differences in SCC of *S. aureus* positive herds and herds tested negative for *S. aureus* were found (as calculated by Student's *t*-test, *P* < 0.05). The BTM SCC was increased (>200,000 cells/ml) in two BTM samples (farms 3 and 16) but represented just in one case (farm 3) an indicator for a herd problem with *S. aureus* (Table 1). Based on their SCC and bacterial count values, the BTM from all other farms could be qualified—according to Austrian milk quality regulation—as so called “S-class-milk” of highest quality (SCC < 250,000 SCC/ml).

**Figure 1:**
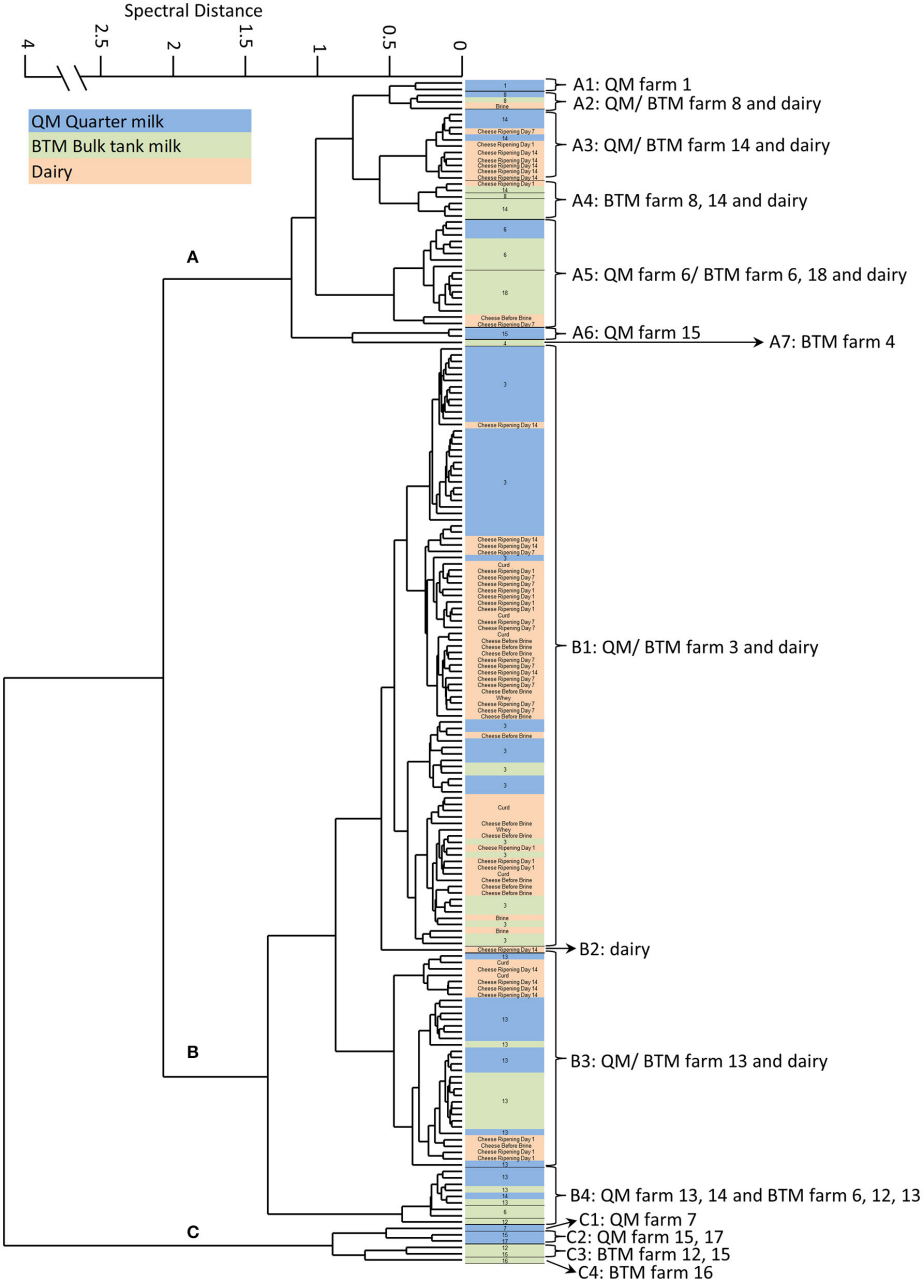
**FTIR spectroscopy-based hierarchical cluster analysis (HCA) of *S. aureus* isolated from quarter milk samples of individual cows (QM), samples from farm bulk tanks (BTM) and different steps of cheese processing and ripening at the dairy plant (Dairy) (for details see Supplementary Table S1)**. The HCA of *S. aureus* spectra data revealed three main clusters, designated **(A-C)**.

The whole collected BTM (5108.70 l) was manufactured into 72 artisan semi-hard cheeses. Samples were taken at defined points throughout the production of these cheeses and analyzed for the presence of Staphylococci. *S. aureus* could be isolated throughout the manufacturing process until day 14 of ripening (see Figure 1, Table 2).

**Table 2:** **Strain characteristics of *S. aureus* isolates according to their FTIR biotype**.

**FTIR biotype**		**QM/ BTM/ Dairy (n)^a^**	***spa* type^b^**	**ST type (CC)^b^**	**CP type**	**Enterotoxin gene profile^b^**	**MRSA**
A	1	QM1 (2)	t524	71 (97)	NT	neg	neg
	2	QM8 (1)/ BTM8 (1)/ Dairy_B (1)	t524	71 (97)	NT	neg	neg
	3	QM14 (4)/ Dairy_R1 (1); Dairy_R7 (1); Dairy_R14 (5)	t044	97 (97)	NT	neg	neg
	4	BTM 8 (1); BTM14 (4)/ Dairy_R1 (1)	t044	97 (97)	NT	neg	neg
	5	QM6 (3)/ BTM6 (3); BTM18 (6)/ Dairy_BB (1); Dairy_R7 (1)	t044	97 (97)	NT	neg	neg
		BTM6 (2); BTM18 (1)	t044	97 (97)	CP5	neg	neg
	6	QM15 (2)	t337	n.d.	CP5	*seg, sei*	neg
	7	BTM4 (1)	t056	101 (Sing.)	NT	neg	neg
B	1	QM3 (39)/ BTM3 (10)/ Dairy_C (8); Dairy_W (1); Dairy_BB (11); Dairy_B (1); Dairy_R1 (9); Dairy_R7 (11); Dairy_R14 (4)	t2953	8 (8)	NT	*sea, sed, sej*	neg
	2	Dairy_R1 (1)	t084	15 (15)	NT	neg	neg
	3	QM13 (14)/ BTM13 (10)/ Dairy_C (2); Dairy_BB (1); Dairy_R1 (3); Dairy_R14 (4)	t2953	8 (8)	NT	*sea, sed, sej*	neg
	4	QM13 (3)/ BTM13 (2)	t2953	8 (8)	NT	*sea, sed, sej*	neg
		BTM6 (2); BTM12 (1)	t2953	8 (8)	NT	neg	neg
		QM14 (1)	t2953	8 (8)	NT	neg	neg
C	1	QM7 (1)	n.d.	n.d.	CP8	*seg, sei*	neg
	2	QM15 (1); QM17 (1)	t529	504 (705)	CP8	*seg, sei*	neg
	3	BTM12 (1)	t529	504 (705)	CP8	*seg, sei*	neg
		BTM15 (1)	t529	504 (705)	CP8	*sea, sed, sej*	neg
	4	BTM16 (1)	n.d.	n.d.	CP8	neg	neg

a Number in brackets indicate number of isolates (n); QM (quarter milk) and BTM (bulk tank milk) numbers are referring to the farm number; Dairy_X refer to the respective cheese production steps (X) at the dairy plant: C (curd), W (whey), BB (before brine), B (brine) and R1, 7, 14 (ripening day 1, 7, 14);

b *Determined for a subset of strains (for details see Supplementary Table S1)*.

### Isolation and identification of staphylococci

In total, 313 presumptive *Staphylococcus* strains were isolated from the whole dairy chain from cow to cheese. All isolates were cultured and first classified with traditional microbiological approaches. Subsequently, FTIR spectroscopy was employed for identification of isolates as outlined in the material and method section. FTIR spectra were recorded from all isolates and spectral reference libraries were used for species identification. sixty percentage (187/313) of the isolates could be assigned to *S. aureus* while 40% (126/313) of the isolates were assigned to other Staphylococcus species. *Staphylococcus chromogenes* (22%) *and Staphylococcus haemolyticus* (18%), followed by *S. pasteuri, S. hominis subsp. Hominis*, and *S. hominis subsp. novobiosepticus*, were the predominant species (data not shown).

From the total of 187 *S. aureus* strains isolated in frame of this study, 64% originate from farm (72 strains from QM and 47 strains from BTM) and 36% from several cheese processing levels at the dairy plant (68 strains). Among the 178 *S. aureus* strains, αß-hemolysis was predominately found (53.44%), followed by isolates showing α-hemolysis (35.06%). 5.75% of the isolates showed ß- or no hemolysis. For clumping factor, 73.60% of all *S. aureus* strains were positive and 26.40% negative. The distribution of coagulase reaction showed 91.01% positive and 8.99% negative tested isolates. Of all strains, 5.62% were coincidently clumping factor negative as well as coagulase negative, but revealed either a typical colony morphology and/or αß-hemolysis. Among strains isolated from the same sample, differences in hemolysis, clumping factors and coagulase reactions were observed. Species identification of all *S. aureus* strains was further confirmed by *nuc* specific PCR. A screening of all *S. aureus* isolates for MRSA, using the BBL CHROMagar MRSA and *mecA* PCR, showed that all isolates are MSSA.

### Population analysis of *S. aureus* strains isolated from the dairy production chain

Chemometrics was used to further analyze FTIR spectral date recorded form all *S. aureus* strains (*n* = 187). Hierarchical cluster analysis (HCA) was employed for gaining insights into the population structure and diversity of strains from the same source and to follow potential transmission routes from the QM samples of individual cows into the cheese processing at the dairy plant. The HCA revealed a high inter farm—and in some cases also intra farm- diversity of *S. aureus* strains (Table 2). As shown in Figure 1, the *S. aureus* strains can be assigned to 3 main clusters. Cluster A (*n* = 42) is dominated by strains from BTM isolated from 6 different farms and also contains several QM strains derived from the aforementioned farms as well as strains isolated from cheese production at the dairy plant (designated as “dairy” strains). Cluster B (*n* = 139) is dominated by strains isolated from QM and from BTM of two farms (farm 3 and 13) and strains isolated during cheese production until day 14 of cheese ripening. Cluster C comprises the smallest amount of isolates (*n* = 6). To the latter cluster only strains are assigned, which have been isolated from farms with low *S. aureus* prevalence rates (1/25 animals at farm 7, 2/22 at farm 15, and 1/5 at farm 17) or from farms with *S. aureus* positive BTM but *S. aureus* negative QM samples (farm 12 and 16; see Table 1). Notable, the *S. aureus* intra farm diversity was higher at farms with low *S. aureus* prevalence rates than at farms with high prevalence rates. At farm 15, the strains derived from QM of two animals tested positive for *S. aureus*, fall into two different clusters; the isolates from one animal are found in cluster A while the isolate from the second animal is found in cluster C (Table 2). In contrast, all strains isolated at farm 3 from QM and BTM were clustering closely together in cluster B1. The prevalence rate of *S. aureus* at the latter farm exceeded 50%. ten out of 17 cows were positive tested for *S. aureus* (Table 1). The HCA also revealed that the same *S. aureus* subtypes found on farm level in QM and BTM samples from farm 3, 6, 8, 13, 14, and 18 are also present in samples derived from the dairy plant at various cheese production steps (Figure 1). Most of the isolates from the cheese production at the dairy plant (45/65) are clustering together with the isolates from farm 3 in cluster B1. Ten isolates from cheese production are assigned to cluster A2, A3, A4, and A5. One isolate from cheese (ripening day 14) represents cluster B2 as a singleton and the remaining ten isolates from the cheese production fall into cluster B3, together with isolates from farm 13. At the latter farm a second *S. aureus* subtype (B4) was found, which was also detected in BTM samples from three other farms but not in any dairy plant samples. Interestingly, strains belonging to cluster C were only isolated at farm level but not from the dairy plant (Supplementary Table S2).

Since capsular polysaccharide expression was shown to be associated with *S. aureus* persistence, we next investigated if the potential for transmission of *S. aureus* from farm to dairy might be linked to capsule production capacities of strains. The latter was tested by employing a previously established artificial neural network (ANN) for capsule type (CP) determination based on FTIR spectral data (Grunert et al., 2013). All cluster B strains were negative for capsule production (NT type) while all cluster C strains were assigned to CP8 (Table 2). A few strains belonging to cluster A were assigned to CP5 but the majority of cluster A strains was negative for capsule production (NT type).

### Molecular subtyping and enterotoxin profiling

To gain further insights into the molecular characteristics of the *S. aureus* strains isolated in frame of this work, *spa* types, and ST types of selected strains from the different FTIR clusters were determined following standard procedures as described in material and methods (see Table 2). Strains clustering in A1 and A2 were assigned to t524 (ST71) while strains from cluster A3, A4, and A5 belong to t044 (ST97) and strain from cluster A6 and A7 to t337 and t056 (ST101), respectively. All strains, except one strain representing cluster B2 as singleton, from cluster B belong to t2953 (ST8). The strain representing cluster B2, which was derived from cheese at ripening day 14, belong to t084 (ST15) while strains from cluster C were assigned to t529 (ST504). Enterotoxin gene profiles of selected strains were determined by multiplex PCR. The vast majority of strains, except two isolates from QM samples of one animal (cluster A6 *seg*/*sei*), from cluster A were negative for the tested enterotoxin genes A, B, C, D, E, G, H, I, and J. All strains tested belonging to cluster B show the combination *sea*/*sed*/*sej*, except two strains from a bulk tank milk, which were negative for all enterotoxin genes tested. From the six strains tested belonging to cluster C, four carry the combination *seg*/*sei*, one the combination *sea*/*sed*/*sej* and one was negative for all enterotoxin genes tested.

## Discussion

This study provides a comprehensive analysis of the potential of primary production as source for *S. aureus* contaminations in the dairy production and processing chain. The dairy chain opens various entrance points for the human pathogen *S. aureus*, including the primary production environment and people involved in the dairy production and processing (Haveri et al., 2008; Johler et al., 2016b). Recent molecular studies as well as reports from food borne dairy associated outbreak linked to *S. aureus* indicate a potential, yet not sufficiently explored, role of dairy cows as entrance point of *S. aureus* into the dairy production chain (Schmid et al., 2009; Hummerjohann et al., 2014; Walcher et al., 2014; Johler et al., 2015). Thus, in the current study, special emphasis was placed on sampling of dairy cows on quarter milk level to follow *S. aureus* over the complete production and processing chain of a semi-hard raw milk cheese manufactured at an alpine dairy—from cow to product.

### Dairy cow—first entrance point of *S. aureus* into the dairy production chain

FTIR analysis of the 187 *S. aureus* strains (119 originating from dairy farms and 68 from cheese production at the dairy plant) isolated in frame of this study, revealed the bovine udder as an important *S. aureus* contamination source for the dairy production chain (Figure 1). Generally, the three major clusters revealed by FTIR are linked to CCs (CC97, CC8, CC705) typically found in bovine *S. aureus* (Smith et al., 2005; Johler et al., 2016b). Only two strains, representing the subclusters A7 and B2 as singeltons, were assigned to the human STs (Table 2). One strain originated from a BTM sample at farm 4 (ST101) and the other from the cheese at day 14 of ripening at the dairy (ST15), suggesting humans as a rather minor *S. aureus* contamination source in the dairy production and processing chain under investigation. *S. aureus* could be isolated and traced throughout the dairy chain from single animals, farm BTM and dairy processing up to 14 days of cheese ripening. Generally, the diversity of strains from primary production (QM and BTM) was greater compared to the diversity of strains from dairy production. Strains from dairy production were found in cluster A and B together with QM and BTM strains, but not in cluster C. Cluster C was restricted to primary production (QM and BTM), indicating that not all dairy cow associated strain are capable to contaminate and persist in the cheese production chain. Indeed, cluster C contains strains belonging to ST504 (CC705, former CC151), a typical bovine associated CC (Herron-Olson et al., 2007; Ben Zakour et al., 2008). The hypothesis that *S. aureus* farm strains differ in their capacities for transfer and persistence in dairy production is fostered by the results from farm 13 samples. Isolates from QM and BTM of farm 13 are grouped in two clusters, namely B3 and B4. In B3 these isolates are intermingled with dairy isolates, pointing toward a contamination of the dairy production by these isolates while cluster B4 is restricted to strains isolated from farm samples (see Table 2). The vast majority of dairy isolates (84%) and QM isolates (79%) belongs to cluster B while BTM isolates were more evenly distributed among the three clusters. A link between QM and dairy isolates was found for strains from 5 farms, although to a different degree. Generally, cluster A strains originated from farms with low *S. aureus* within herd prevalence (one or two *S. aureus* positive animals) while the majority of cluster B farm strains originated from farm 3 and farm 13, two herds with high *S. aureus* within herd prevalence (59% farm 3 and 46% farm 13). Generally, the *S. aureus* prevalence in dairy cattle herds found in our study (Table 1) is comparable to the prevalence rates from other studies, reporting prevalence rates of 0.7–6% for low prevalence herds (LP) and 28–62% for high prevalence herds (HP) (Cremonesi et al., 2015). We also found in our study all classical bovine associated genotypes (CC complexes, *spa* types) reported recently in a European survey (Boss et al., 2016), including the intercontinental CC 97 (Smith et al., 2005). Thus, it could be assumed that the results from our survey are representative for dairy production and processing. For instance, cluster A strains could be assigned to the genotypes CLF, CLI, and CLR, cluster B strains to the genotype CLB and cluster C strains to the CLC, all genotypes frequently present in milk samples from inframammary infections surveyed in a recent European study, which included 12 different countries (Boss et al., 2016).

### Is specific bovine udder adapted *S. aureus* subtype persistent in the cheese production environment?

Although the milk quantity from farm 3 and farm 13 (Table 1) delivered to the dairy plant accounted only for 4% of the total milk volume used for the production of the cheeses, farm 3 and farm 13 *S. aureus* strains achieved the most successful entry of *S. aureus* from farm to dairy. Notable, all farm 3 and farm 13 strains belong to cluster B, a cluster in which almost all strains analyzed were assigned to t2953, regardless of their origin. t2953, is the bovine associated *spa* type of CC8 that is known for its contagiosity and high within herd prevalence (Cremonesi et al., 2015; Boss et al., 2016) and, as shown in our current study, is also successfully transferred to the cheese production and processing from the bovine udder.

Although we found some dairy strains in cluster A, most of the dairy isolates were assigned together with CC 8 farm strains to cluster B. Thus, it is tempting to speculate that the CC8 farm strains belonging to cluster B are well adapted to the cheese production environment. In particular, their capacity to adhere and form biofilms on the surface of milk processing equipment at dairy plants could contribute to be a source of *S. aureus* contamination of dairy production (Sharma and Anand, 2002; Gutiérrez et al., 2012).

Notable, some of the cluster A and all of the cluster C farm strains—but none of the cluster B strains—showed expression of capsule polysaccharides. All cluster C strains belong to CP8 while the CP positive strains in cluster A were assigned to CP5. It was shown that the encapsulated *S. aureus* strains CP5, CP8, and the non-encapsulated strains can be reliable discriminated by FTIR spectroscopy since the main discriminatory spectral features are primarily based on bacterial surface glycopolymers including capsular polysaccharides (Grunert et al., 2013; Johler et al., 2016a). In particular, our study confirmed the high prevalence of non-encapsulated *S. aureus* strains derived from bovine mastitis, which underscores the importance of losing CP expression to be expected a key *S. aureus* feature associated with persistence (Sordelli et al., 2000; Tuchscherr et al., 2010). The absence of CP expression was shown previously to elevate the exposure of surface-associated adhesins, supporting bacterial adhesion to host cells (Pöhlmann-Dietze et al., 2000; Risley et al., 2007).

None of the dairy strains showed CP expression, which may indicate that similar mechanism allow the successfully entrance and persistence of non-encapsulated strains in the dairy processing. However, further studies systematically addressing the capsule production of dairy *S. aureus* strains will be necessary to elucidate a potential function of capsule production repression in dairy production and processing environments.

Molecular typing and enterotoxin gene profiling revealed that cluster B strains show genetic characteristics (t2953, ST8, and enterotoxin genes *sea*/*sed*/*sej*) of the recently described *S. aureus* genotype B (Fournier et al., 2008; Cremonesi et al., 2015). Genotype B has been reported to be linked to high within herd prevalence und frequent intramammary infections (Fournier et al., 2008; Graber et al., 2009), which is in accordance to the results from our current study. Cluster B strains originated from two farms with high *S. aureus* prevalence (farm 3 56, farm 13 46%). It is therefore tempting to speculate that cluster B strains are indeed representatives of genotype B, which can easily be detected and traced by FTIR spectroscopy as a cost effective high throughput metabolic fingerprinting method.

As shown in our current work, strains with genotype B characteristics are successfully transmitted from cows to the dairy production. Since these strains pose a potential health risk for the consumer, efficient detection and monitoring of this specific *S. aureus* subtypes would not only be important in the light of cow health, as has been recommended by Fournier et al. (2008), but would also be important from a food safety perspective. For instance, very recently a strain with genotype B characteristics isolated from soft cheese was linked to a food borne outbreak at a boarding school (Johler et al., 2015) and a recenty study of Hummerjohann et al. (2014) revealed that gentype B is the predominant *S. aureus* subtype in semi-hard cheeses made from raw milk.

### Zoonotic potential of bovine *S. aureus* transmitted into dairy chain

The strains belonging to cluster B (ST2945), which have been most successfully transmitted to the dairy, possess the genes for the enterotoxins SEA, SED, and SEJ that are known for their toxigenic potential for humans. SEA and SED are the major toxins linked to human foodborne outbreaks, including dairy product associated ones (Asao et al., 2003; Schmid et al., 2009; Argudín et al., 2010; Johler et al., 2015). Notable, a SEA and SAD strain from QM showing the same *spa* type (t2953) as our cluster B strains was recently identified as cause of dairy product related outbreak in Lower Austria (Schmid et al., 2009). Generally, strains from cluster A were negative for enterotoxin genes. Only two isolates from one animal of farm 15 were positive for SEG and SEI. However, these strains clustered separately from other strains (subcluster A6) and not together with any dairy strains. Thus, it could be assumed that the zoonotic potential of cluster A strains is rather low and emphasis should be placed on the early detection and prevention of the transmission of cluster B strains to the dairy production.

## Conclusion

In conclusion, our works highlights the importance of effective hygienic measures on farm level, reemphasizing that food safety starts with the healthy animal. As revealed by FTIR spectroscopy, *S. aureus* can effectively enter the dairy production chain via contaminated milk of cows with subclinical *S. aureus* intramammary infections. Certain bovine *S. aureus* subtypes, showing characteristics of the recently described genotype B (Fournier et al., 2008; Graber et al., 2009), appeared to be better equipped than others for successful transmission into the dairy production and processing. Further studies will be necessary to elucidate the factors allowing these specific *S. aureus* subtypes to conquest the dairy production chain.

## Author contributions

ME, MW, and BS have conceptualized and supervised the study. JK, BS, and GW carried out the farm and dairy plant sampling. OB organized and supervised the farm and dairy plant sampling. JK, BS, and MG performed the strain characterization. FTIR analysis and molecular subtyping was done by TG, MF, JK, BS, and MG. Wrote and revised the paper: JK, ME, BS, TG, and MW. ME, acted as overall study director.

### Conflict of interest statement

The authors declare that the research was conducted in the absence of any commercial or financial relationships that could be construed as a potential conflict of interest.
