# Supplementary material for: Staphylococcus aureus Entrance into the Dairy Chain: Tracking S. aureus from Dairy Cow to Cheese
Source: Front Microbiol. 2016 Oct 13;7:1603. doi: 10.3389/fmicb.2016.01603 (PMC5061776; doi:10.3389/fmicb.2016.01603)
Supplement: Supplementary file 1 [file Table1.PDF]

Suppl. Table S1

Staphylococcus aureus entrance into the dairy chain: Tracking *S. aureus* from dairy cow to cheese

Judith Kümmel, Beatrix Stessl, Monika Gonano, Georg Walcher, Othmar Bereuter, Martina Fricker, Tom Grunert, Martin Wagner, Monika Ehling-Schulz

Overview of sources, *spa*-type, ST type and clonal complex (CC) assignment, capsular serotype, and enterotoxin gene profile of all *S. aureus* strains used in this study.

FTIR maincluster	FTIR subcluster	Sample type	*spa*-type	ST type (CC)	Capsular serotype	Enterotoxin gene profile
A	A1	QM Farm1 Animal 12	n.d.	n.d.	NT	n.d.
		QM Farm1 Animal 12	t524	71 (97)	NT	neg
	A2	QM Farm 8 Animal 13	t524	71 (97)	NT	neg
		BTM Farm 8	t524	71 (97)	NT	neg
		Dairy Brine	t524	71 (97)	NT	neg
	A3	QM Farm 14 Animal 6	n.d.	n.d.	NT	n.d.
		QM Farm 14 Animal 17	t044	97 (97)	NT	neg
		QM Farm 14 Animal 6	t044	97 (97)	NT	neg
		Dairy Cheese Ripening Day 7	n.d.	n.d.	NT	neg
		QM Farm 14 Animal 6	n.d.	n.d.	NT	n.d.
		Dairy Cheese Ripening Day 1	n.d.	n.d.	NT	neg
		Dairy Cheese Ripening Day 14	n.d.	n.d.	NT	n.d.
		Dairy Cheese Ripening Day 14	n.d.	n.d.	NT	n.d.
		Dairy Cheese Ripening Day 14	n.d.	n.d.	NT	n.d.
		Dairy Cheese Ripening Day 14	n.d.	n.d.	NT	n.d.
		Dairy Cheese Ripening Day 14	n.d.	n.d.	NT	n.d.
	A4	Dairy Cheese Ripening Day 1	t044	97 (97)	NT	neg
		BTM Farm 14	t044	97 (97)	NT	neg
		BTM Farm 8	n.d.	n.d.	NT	neg
		BTM Farm 14	n.d.	n.d.	NT	n.d.
		BTM Farm 14	t044	97 (97)	NT	neg
		BTM Farm 14	t044	97 (97)	NT	neg
	A5	QM Farm 6 Animal 8	n.d.	n.d.	NT	n.d.
		QM Farm 6 Animal 14	n.d.	n.d.	NT	neg
		QM Farm 6 Animal 14	t044	97 (97)	NT	neg
		BTM Farm 6	t044	97 (97)	ND	neg
		BTM Farm 6	t044	97 (97)	ND	neg
		BTM Farm 6	t044	97 (97)	ND	neg
		BTM Farm 6	t044	97 (97)	CP5	neg
		BTM Farm 6	t044	97 (97)	CP5	neg
		BTM Farm 18	n.d.	n.d.	CP5	n.d.
		BTM Farm 18	t044	97 (97)	NT	neg
		BTM Farm 18	t044	97 (97)	ND	neg
		BTM Farm 18	t044	97 (97)	NT	neg
		BTM Farm 18	t044	97 (97)	ND	neg
		BTM Farm 18	t044	97 (97)	ND	neg
		BTM Farm 18	t044	97 (97)	NT	neg
		Dairy Cheese Before Brine	t044	97 (97)	NT	neg
		Dairy Cheese Ripening Day 7	t044	97 (97)	NT	neg
	A6	QM Farm 15 Animal 17	t337	n.d.	CP5	*seg, sei*
		QM Farm 15 Animal 17	t337	n.d.	CP5	*seg, sei*
	A7	BTM Farm 4	t056	101 (Singular)	NT	neg
B	B1	QM Farm 3 Animal 13	n.d.	n.d.	NT	*sea. sed. sei*
		QM Farm 3 Animal 14	n.d.	n.d.	NT	*sea. sed. sei*
		QM Farm 3 Animal 14	t2953	8 (8)	NT	*sea. sed. sei*
		QM Farm 3 Animal 10	t2953	8 (8)	NT	*sea. sed. sei*
		QM Farm 3 Animal 15	n.d.	n.d.	NT	*sea. sed. sei*
		QM Farm 3 Animal 17	n.d.	n.d.	NT	*sea. sed. sei*
		QM Farm 3 Animal 6	n.d.	n.d.	NT	*sea. sed. sei*
		QM Farm 3 Animal 5	t2953	8 (8)	NT	*sea. sed. sei*
		QM Farm 3 Animal 14	t2953	8 (8)	NT	*sea. sed. sei*
		QM Farm 3 Animal 14	t2953	8 (8)	NT	*sea. sed. sei*
		QM Farm 3 Animal 16	n.d.	n.d.	NT	*sea. sed. sei*
		QM Farm 3 Animal 16	n.d.	n.d.	NT	*sea. sed. sei*
		Dairy Cheese Ripening Day 14	n.d.	n.d.	NT	n.d.
		QM Farm 3 Animal 14	n.d.	n.d.	NT	*sea. sed. sei*
		QM Farm 3 Animal 15	n.d.	n.d.	NT	*sea. sed. sei*
		QM Farm 3 Animal 15	n.d.	n.d.	NT	*sea. sed. sei*
		QM Farm 3 Animal 16	t2953	8 (8)	NT	*sea. sed. sei*
		QM Farm 3 Animal 14	t2953	8 (8)	NT	*sea. sed. sei*
		QM Farm 3 Animal 9	n.d.	n.d.	NT	*sea. sed. sei*
		QM Farm 3 Animal 1	t2953	8 (8)	NT	*sea. sed. sei*
		QM Farm 3 Animal 15	n.d.	n.d.	NT	*sea. sed. sei*
		QM Farm 3 Animal 1	t2953	8 (8)	NT	*sea. sed. sei*
		QM Farm 3 Animal 10	n.d.	n.d.	NT	*sea. sed. sei*
		QM Farm 3 Animal 5	t2953	8 (8)	NT	*sea. sed. sei*
		QM Farm 3 Animal 15	n.d.	n.d.	NT	*sea. sed. sei*
		QM Farm 3 Animal 17	n.d.	n.d.	NT	*sea. sed. sei*
		QM Farm 3 Animal 9	n.d.	n.d.	NT	*sea. sed. sei*
		QM Farm 3 Animal 13	n.d.	n.d.	NT	*sea. sed. sei*
		QM Farm 3 Animal 15	n.d.	n.d.	NT	*sea. sed. sei*
		QM Farm 3 Animal 1	n.d.	n.d.	NT	*sea. sed. sei*
		Dairy Cheese Ripening Day 14	n.d.	n.d.	NT	n.d.
		Dairy Cheese Ripening Day 14	n.d.	n.d.	NT	n.d.
		Dairy Cheese Ripening Day 7	n.d.	n.d.	NT	*sea. sed. sei*
		QM Farm 3 Animal 10	t2953	8 (8)	NT	*sea. sed. sei*
		Dairy Curd	n.d.	n.d.	NT	*sea. sed. sei*
		Dairy Cheese Ripening Day 1	n.d.	n.d.	NT	*sea. sed. sei*
		Dairy Cheese Ripening Day 7	n.d.	n.d.	NT	*sea. sed. sei*
		Dairy Cheese Ripening Day 7	n.d.	n.d.	NT	*sea. sed. sei*
		Dairy Cheese Ripening Day 1	n.d.	n.d.	NT	*sea. sed. sei*
		Dairy Cheese Ripening Day 1	n.d.	n.d.	NT	*sea. sed. sei*
		Dairy Cheese Ripening Day 1	n.d.	n.d.	NT	*sea. sed. sei*
		Dairy Cheese Ripening Day 1	n.d.	n.d.	NT	*sea. sed. sei*
		Dairy Curd	t2953	8 (8)	NT	*sea. sed. sei*
		Dairy Cheese Ripening Day 7	n.d.	n.d.	NT	*sea. sed. sei*
		Dairy Cheese Ripening Day 7	t2953	8 (8)	NT	*sea. sed. sei*
		Dairy Curd	n.d.	n.d.	NT	*sea. sed. sei*
		Dairy Cheese Before Brine	n.d.	n.d.	NT	*sea. sed. sei*
		Dairy Cheese Before Brine	n.d.	n.d.	NT	*sea. sed. sei*
		Dairy Cheese Before Brine	n.d.	n.d.	NT	*sea. sed. sei*
		Dairy Cheese Ripening Day 7	n.d.	n.d.	NT	*sea. sed. sei*
		Dairy Cheese Ripening Day 7	n.d.	n.d.	NT	*sea. sed. sei*
		Dairy Cheese Ripening Day 14	t2953	8 (8)	NT	*sea. sed. sei*
		Dairy Cheese Ripening Day 7	n.d.	n.d.	NT	*sea. sed. sei*
		Dairy Cheese Ripening Day 7	n.d.	n.d.	NT	*sea. sed. sei*
		Dairy Cheese Before Brine	t2953	8 (8)	NT	*sea. sed. sei*
		Dairy Whey	t2953	8 (8)	NT	*sea. sed. sei*
		Dairy Cheese Ripening Day 7	t2953	8 (8)	NT	*sea. sed. sei*
		Dairy Cheese Ripening Day 7	t2953	8 (8)	NT	*sea. sed. sei*
		Dairy Cheese Before Brine	t2953	8 (8)	NT	*sea. sed. sei*
		QM Farm 3 Animal 14	n.d.	n.d.	NT	*sea. sed. sei*
		QM Farm 3 Animal 16	n.d.	n.d.	NT	*sea. sed. sei*
		Dairy Cheese Before Brine	n.d.	n.d.	NT	*sea. sed. sei*
		QM Farm 3 Animal 1	t2953	8 (8)	NT	*sea. sed. sei*
		QM Farm 3 Animal 5	n.d.	n.d.	NT	*sea. sed. sei*
		QM Farm 3 Animal 5	n.d.	n.d.	NT	*sea. sed. sei*
		QM Farm 3 Animal 5	n.d.	n.d.	NT	*sea. sed. sei*
		BTM Farm 3	t2953	8 (8)	NT	*sea. sed. sei*
		BTM Farm 3	t2953	8 (8)	NT	*sea. sed. sei*
		QM Farm 3 Animal 5	n.d.	n.d.	NT	*sea. sed. sei*
		QM Farm 3 Animal 10	t2953	8 (8)	NT	*sea. sed. sei*
		QM Farm 3 Animal 5	n.d.	n.d.	NT	*sea. sed. sei*
		Dairy Curd	n.d.	n.d.	NT	*sea. sed. sei*
		Dairy Curd	t2953	8 (8)	NT	*sea. sed. sei*
		Dairy Curd	t2953	8 (8)	NT	*sea. sed. sei*
		Dairy Curd	n.d.	n.d.	NT	*sea. sed. sei*
		Dairy Cheese Before Brine	n.d.	n.d.	NT	*sea. sed. sei*
		Dairy Whey	n.d.	n.d.	NT	*sea. sed. sei*
		Dairy Cheese Before Brine	n.d.	n.d.	NT	*sea. sed. sei*
		BTM Farm 3	t2953	8 (8)	NT	*sea. sed. sei*
		Dairy Cheese Ripening Day 1	t2953	8 (8)	NT	*sea. sed. sei*
		BTM Farm 3	n.d.	n.d.	NT	*sea. sed. sei*
		Dairy Cheese Ripening Day 1	n.d.	n.d.	NT	*sea. sed. sei*
		Dairy Cheese Ripening Day 1	t2953	8 (8)	NT	*sea. sed. sei*
		Dairy Curd	t2953	8 (8)	NT	*sea. sed. sei*
		Dairy Cheese Before Brine	n.d.	n.d.	NT	*sea. sed. sei*
		Dairy Cheese Before Brine	n.d.	n.d.	NT	*sea. sed. sei*
		Dairy Cheese Before Brine	n.d.	n.d.	NT	*sea. sed. sei*
		BTM Farm 3	n.d.	n.d.	NT	*sea. sed. sei*
		BTM Farm 3	t2953	8 (8)	NT	*sea. sed. sei*
		BTM Farm 3	n.d.	n.d.	NT	*sea. sed. sei*
		Dairy Brine	t2953	8 (8)	NT	*sea. sed. sei*
		BTM Farm 3	t2953	8 (8)	NT	*sea. sed. sei*
		Dairy Cheese Ripening Day 1	t2953	8 (8)	NT	*sea. sed. sei*
		BTM Farm 3	n.d.	n.d.	NT	*sea. sed. sei*
		BTM Farm 3	t2953	8 (8)	NT	*sea. sed. sei*
	B2	Dairy Cheese Ripening Day 14	t084	15 (15)	NT	neg
	B3	QM Farm 13 Animal 3	n.d.	n.d.	NT	*sea. sed. sei*
		Dairy Curd	n.d.	n.d.	NT	*sea. sed. sei*
		Dairy Cheese Ripening Day 14	n.d.	n.d.	NT	n.d.
		Dairy Curd	n.d.	n.d.	NT	*sea. sed. sei*
		Dairy Cheese Ripening Day 14	n.d.	n.d.	NT	n.d.
		Dairy Cheese Ripening Day 14	n.d.	n.d.	NT	n.d.
		Dairy Cheese Ripening Day 14	n.d.	n.d.	NT	n.d.
		QM Farm 13 Animal 3	n.d.	n.d.	NT	*sea. sed. sei*
		QM Farm 13 Animal 5	n.d.	n.d.	NT	*sea. sed. sei*
		QM Farm 13 Animal 7	n.d.	n.d.	NT	*sea. sed. sei*
		QM Farm 13 Animal 11	n.d.	n.d.	NT	n.d.
		QM Farm 13 Animal 11	n.d.	n.d.	NT	*sea. sed. sei*
		QM Farm 13 Animal 3	t2953	8 (8)	NT	*sea. sed. sei*
		QM Farm 13 Animal 12	n.d.	n.d.	NT	*sea. sed. sei*
		BTM Farm 13	n.d.	n.d.	NT	*sea. sed. sei*
		QM Farm 13 Animal 5	n.d.	n.d.	NT	n.d.
		QM Farm 13 Animal 10	n.d.	n.d.	NT	*sea. sed. sei*
		QM Farm 13 Animal 11	n.d.	n.d.	NT	n.d.
		QM Farm 13 Animal 5	t2953	8 (8)	NT	*sea. sed. sei*
		BTM Farm 13	n.d.	n.d.	NT	*sea. sed. sei*
		BTM Farm 13	n.d.	n.d.	NT	*sea. sed. sei*
		BTM Farm 13	n.d.	n.d.	NT	*sea. sed. sei*
		BTM Farm 13	n.d.	n.d.	NT	*sea. sed. sei*
		BTM Farm 13	n.d.	n.d.	NT	*sea. sed. sei*
		BTM Farm 13	t2953	8 (8)	NT	*sea. sed. sei*
		BTM Farm 13	t2953	8 (8)	NT	*sea. sed. sei*
		BTM Farm 13	n.d.	n.d.	NT	*sea. sed. sei*
		BTM Farm 13	n.d.	n.d.	NT	*sea. sed. sei*
		QM Farm 13 Animal 12	t2953	8 (8)	NT	*sea. sed. sei*
		Dairy Cheese Ripening Day 1	n.d.	n.d.	NT	*sea. sed. sei*
		Dairy Cheese Before Brine	t2953	8 (8)	NT	*sea. sed. sei*
		Dairy Cheese Ripening Day 1	t2953	8 (8)	NT	*sea. sed. sei*
		Dairy Cheese Ripening Day 1	t2953	8 (8)	NT	*sea. sed. sei*
		QM Farm 13 Animal 11	n.d.	n.d.	NT	*sea, sed, sej*
	B4	QM Farm 13 Animal 3	n.d.	n.d.	NT	*sea. sed. sei*
		QM Farm 13 Animal 5	n.d.	n.d.	NT	*sea. sed. sei*
		QM Farm 13 Animal 3	t2953	8 (8)	NT	*sea. sed. sei*
		BTM Farm 13	t2953	8 (8)	NT	*sea. sed. sei*
		QM Farm 14 Animal 17	n.d.	n.d.	NT	neg
		BTM Farm 13	n.d.	n.d.	NT	*sea. sed. sei*
		BTM Farm 6	n.d.	n.d.	NT	neg
		BTM Farm 6	n.d.	n.d.	NT	neg
		BTM Farm 12	n.d.	n.d.	NT	neg
C	C1	QM Farm 7 Animal 1	n.d.	n.d.	CP8	*seg, sei*
	C2	QM Farm 15 Animal 20	t529	n.d.	CP8	*seg. sei*
		QM Farm 17 Animal 5	t529	n.d.	CP8	*seg. sei*
	C3	BTM Farm12	t529	504 (705)	CP8	seg, sei
		BTM Farm 15	t529	504 (705)	CP8	sea, sed, sej
	C4	BTM Farm 16	n.d.	n.d.	CP8	neg

QM (quarter milk), BTM (bulk tank milk), clonal complex (CC), capsular polysaccharide (CP), Non-typeable (NT), not determinable (ND), not determined (n.d.).
