# Supplementary material for: Staphylococcus aureus Entrance into the Dairy Chain: Tracking S. aureus from Dairy Cow to Cheese
Source: Front Microbiol. 2016 Oct 13;7:1603. doi: 10.3389/fmicb.2016.01603 (PMC5061776; doi:10.3389/fmicb.2016.01603)
Supplement: Supplementary file 2 [file Table2.PDF]

Suppl. Table S2

Staphylococcus aureus entrance into the dairy chain: Tracking *S. aureus* from dairy cow to cheese

Judith Kümmel, Beatrix Stessl, Monika Gonano, Georg Walcher, Othmar Bereuter, Martina Fricker, Tom Grunert, Martin Wagner, Monika Ehling-Schulz

Strain characteristics of *S. aureus* isolates according to their production step.

Farm and dairy production (n)		*spa* type[a] (n)	ST type (CC)[a]	CP type	Enterotoxin gene profile[a]	MRSA	FTIR biotype (n)
Quarter milk (72)		t524 (3)	71 (97)	NT	neg	neg	A1 (2); A2 (1)
		t044 (7)	97 (97)	NT	neg	neg	A3 (4); A5 (3)
		t337 (2)	n.d.	CP5	*seg, sei*	neg	A6 (2)
		t2953 (56)	8 (8)	NT	*sea, sed, sej*	neg	B1 (39); B3 (14); B4 (3)
		t2953 (1)	8 (8)	NT	neg	neg	B4 (1)
		n.d.	n.d.	CP8	*seg, sei*	neg	C1 (1)
		t529 (2)	504 (705)	CP8	*seg, sei*	neg	C2 (2)
Bulk tank milk (47)		t524 (1)	71 (97)	NT	neg	neg	A2 (1)
		t044 (14)	97 (97)	NT	neg	neg	A4 (5); A5 (9)
		t044 (3)	97 (97)	CP5	neg	neg	A5 (3)
		t056 (1)	101 (Sing.)	NT	neg	neg	A7 (1)
		t2953 (22)	8 (8)	NT	*sea, sed, sej*	neg	B1 (10); B3 (10); B4 (2)
		t2953 (3)	8 (8)	NT	neg	neg	B4 (3)
		t529 (1)	504 (705)	CP8	*seg, sei*	neg	C3 (1)
		t529 (1)	504 (705)	CP8	*sea, sed, sej*	neg	C3 (1)
		n.d.	n.d.	CP8	neg	neg	C4 (1)
Dairy (68)	Curd (10)	t2953 (10)	8 (8)	NT	*sea, sed, sej*	neg	B1 (8); B3 (2)
	Whey (2)	t2953 (2)	8 (8)	NT	*sea, sed, sej*	neg	B1 (2)
	Cheese Before Brine (13)	t044 (1)	97 (97)	NT	neg	neg	A5 (1)
		t2953 (12)	8 (8)	NT	*sea, sed, sej*	neg	B1 (11); B3 (1)
	Brine (2)	t524 (1)	71 (97)	NT	neg	neg	A2 (1)
		t2953 (1)	8 (8)	NT	*sea, sed, sej*	neg	B1 (1)
	Cheese Ripening Day 1 (14)	t044 (2)	97 (97)	NT	neg	neg	A3 (1); A4 (1)
		t2953 (12)	8 (8)	NT	*sea, sed, sej*	neg	B1 (9); B3 (3)
	Cheese Ripening Day 7 (13)	t044 (2)	97 (97)	NT	neg	neg	A3 (1); A5 (1)
		t2953 (11)	8 (8)	NT	*sea, sed, sej*	neg	B1 (11)
	Cheese Ripening Day 14 (14)	t044 (5)	97 (97)	NT	neg	neg	A3 (5)
		t2953 (8)	8 (8)	NT	*sea, sed, sej*	neg	B1 (4); B3 (4)
		t084 (1)	15 (15)	NT	neg	neg	B2 (1)

[a]Determined for a subset of strains; (n) number of isolates, (CC) clonal complex.
